# Crystal structure of 2,2,3,3-tetra­methyl-1,1,1,4,4,4-hexa­phenyl­tetra­germane

**DOI:** 10.1107/S160053681402501X

**Published:** 2014-11-21

**Authors:** Kirill V. Zaitsev, Sergey S. Karlov, Galina S. Zaitseva, Ali Alizade, Yuri L. Slovokhotov

**Affiliations:** a Department of Chemistry, Moscow State University, 119991 Moscow, Russian Federation; b Moscow State University (Baku Branch), AZ1144 Universitet st. 1, Khojasan, Binagadi, Baku, Azerbaijan

**Keywords:** crystal structure, organo­tetra­germane, zigzag backbone, layered structure

## Abstract

The mol­ecule of the title compound, C_40_H_42_Ge_4_, lies with its central Ge—Ge bond on an inversion centre giving rise to a zigzag backbone of four tetra­hedrally coordinated Ge atoms. The symmetrically independent Ge—Ge bonds are slightly shorter than in other organo­tetra­germanes whereas the Ge—C_Ph_ (Ph = phen­yl) and Ge—C_Me_ (Me = meth­yl) distances have their usual values. In the crystal, (010) layers of Ph_6_Me_4_Ge_4_ mol­ecules with a parallel orientation of the Ge_4_ backbone exist, held together by van der Waals forces only. Main bond lengths in organo-substituted oligogermanes are compared.

## Related literature   

A search for ‘organic electronics’ materials in systems of conjugated C—C bonds (Kobayashi *et al.*, 2011 ▶) was recently extended to organometallic mol­ecules containing chains of atoms such as Ge, Si, or Sn (Marschner & Hlina, 2013 ▶). The established routines used to obtain oligogermanes *via* hydro­germolysis or the reaction of germyllithium reagents with germanium halogenides (Amadoruge & Weinert, 2008 ▶) may give rise to unexpected by-products due to side reactions. As a part of our studies of the chemistry of oligogermanium compounds (Zaitsev *et al.*, 2012 ▶, 2013 ▶, 2014 ▶), the title compound was obtained as a by-product. For related crystal structures of organo­tetra­germanes, see: Roller *et al.* (1986 ▶); Dräger & Simon (1986 ▶); Wagner *et al.* (2009 ▶); Amadoruge *et al.* (2010 ▶).
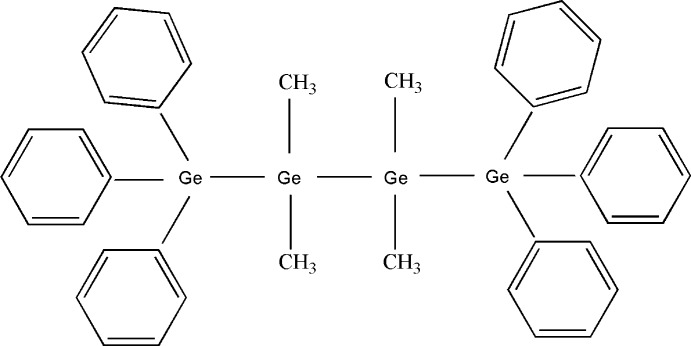


## Experimental   

### Crystal data  

C_40_H_42_Ge_4_
*M* *_r_* = 813.10
Monoclinic, 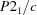

*a* = 9.6402 (5) Å
*b* = 13.6386 (6) Å
*c* = 14.0503 (7) Å
β = 104.560 (1)°
*V* = 1787.99 (15) Å^3^
*Z* = 2
Mo *K*α radiation
μ = 3.36 mm^−1^
*T* = 120 K
0.55 × 0.53 × 0.11 mm

### Data collection  

Bruker APEXII CCD diffractometer
Absorption correction: multi-scan (*SADABS*; Bruker, 2008 ▶) *T*_min_ = 0.391, *T*_max_ = 0.701
22283 measured reflections
5210 independent reflections
4222 reflections with *I* > 2σ(*I*)
*R*_int_ = 0.039

### Refinement  

*R*[*F* ^2^ > 2σ(*F* ^2^)] = 0.030
*wR*(*F* ^2^) = 0.066
*S* = 1.05
5210 reflections
202 parameters
H-atom parameters constrained
Δρ_max_ = 0.62 e Å^−3^
Δρ_min_ = −0.39 e Å^−3^

### 

Data collection: *APEX2* (Bruker, 2008 ▶); cell refinement: *SAINT* (Bruker, 2008 ▶); data reduction: *SAINT*; program(s) used to solve structure: *SHELXTL* (Sheldrick, 2008 ▶); program(s) used to refine structure: *SHELXTL*; molecular graphics: *SHELXTL*; software used to prepare material for publication: *SHELXTL*.

## Supplementary Material

Crystal structure: contains datablock(s) global, I. DOI: 10.1107/S160053681402501X/wm5086sup1.cif

Structure factors: contains datablock(s) I. DOI: 10.1107/S160053681402501X/wm5086Isup2.hkl

**Fig. 1.**
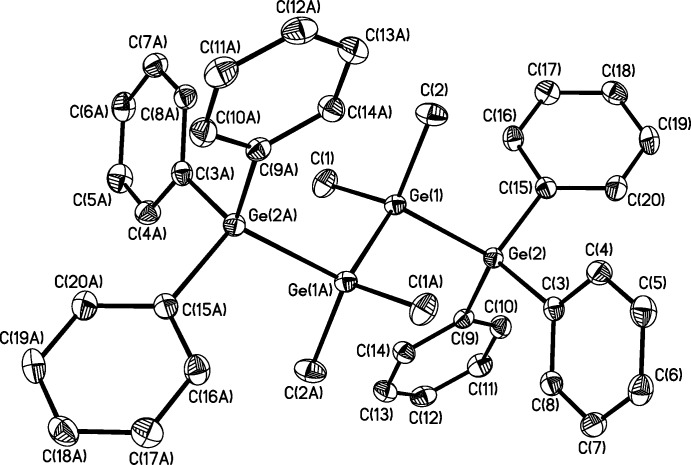
A view of the title molecule showing the atom-numbering scheme. Displacement ellipsoids are drawn at the 50% probability level. Hydrogen atoms are omitted for clarity. [Symmetry operator (A) -x +1, -y+1, -z+1.]

Supporting information file. DOI: 10.1107/S160053681402501X/wm5086Isup3.cml

x y z . DOI: 10.1107/S160053681402501X/wm5086fig1.tif
A view of the title mol­ecule showing the atom-numbering scheme. Displacement ellipsoids are drawn at the 50% probability level. Hydrogen atoms are omitted for clarity. [Symmetry operator (A) −*x* + 1, −*y* + 1, −*z* + 1.]

CCDC reference: 1034201

Additional supporting information:  crystallographic information; 3D view; checkCIF report

## Figures and Tables

**Table 1:** Main bond lengths () in organo-substituted oligogermanes Me is CH_3_, Ms is Me_3_Si and Tol is *p*-C_6_H_4_Me.

Compound	GeGe_periph_	GeGe_central_	GeC_Ph_	GeC_Me_
Ph_3_GeMe_2_GeGeMe_2_GePh_3_ *^*a*^*	2.4361(3)	2.4276(4)	1.961	1.965
Ms_3_GeMe_2_GeGeMe_2_GeMs_3_ *^*b*^*	2.441	2.442		1.967
Tol_3_GeGePh_2_GePh_2_GeTol_3_ *^*c*^*	2.443	2.457	1.973	
Ph_3_GeGePh_2_GePh_2_GePh_3_ *^*d*^*	2.464	2.461	1.969	
Ph_3_GeGeMe_2_GePh_3_ *^*e*^*	2.429		1.957	1.944

References: (*a*) This work; (*b*) Wagner *et al.* (2009 ▶); (*c*) Amadoruge *et al.* (2010 ▶); (*d*) Roller *et al.* (1986 ▶); (*e*) Drger Simon (1986 ▶).

## supplementary crystallographic information

### S1. Experimental

The title compound was obtained in trace amounts from the reaction of two equivalents of Ph_3_GeLi (generated *in situ* from Ph_3_GeH and *n*-BuLi at room temperature in Et_2_O) with Me_2_GeCl_2_ in diethyl ether. The main compound isolated from the mother liquor is the known trigermane Ph_3_GeGeMe_2_GePh_3_ (Dräger & Simon, 1986). Solvent-free crystals of the title compound suitable for X-Ray analysis were recrystallized from toluene at room temperature.

### S2. Refinement

The positions of hydrogen atoms were revealed in difference Fourier maps. For the refinement, they were positioned with idealized geometry and were refined with C—H = 0.95 Å and *U*_eq_(H) = 1.2 *U*_eq_(C) for H atoms attached to aromatic carbon atoms and with C—H = 0.98 Å and *U*_eq_(H) = 1.5*U*_eq_(C) for methyl H atoms. The latter were also allowed for free rotation.

### Crystal data

**Table:**

C_40_H_42_Ge_4_	*F*(000) = 820
*M**_r_* = 813.10	*D*_x_ = 1.510 Mg m^−^^3^
Monoclinic, *P*2_1_/*c*	Mo *K*α radiation, λ = 0.71073 Å
Hall symbol: -P 2ybc	Cell parameters from 7722 reflections
*a* = 9.6402 (5) Å	θ = 2.2–32.5°
*b* = 13.6386 (6) Å	µ = 3.36 mm^−^^1^
*c* = 14.0503 (7) Å	*T* = 120 K
β = 104.560 (1)°	Prism, colourless
*V* = 1787.99 (15) Å^3^	0.55 × 0.53 × 0.11 mm
*Z* = 2	

### Data collection

**Table:**

Bruker APEXII CCD diffractometer	5210 independent reflections
Radiation source: fine-focus sealed tube	4222 reflections with *I* > 2σ(*I*)
Graphite monochromator	*R*_int_ = 0.039
φ and ω scans	θ_max_ = 30.0°, θ_min_ = 2.1°
Absorption correction: multi-scan (*SADABS*; Bruker, 2008)	*h* = −13→13
*T*_min_ = 0.391, *T*_max_ = 0.701	*k* = −19→19
22283 measured reflections	*l* = −19→19

### Refinement

**Table:**

Refinement on *F*^2^	Secondary atom site location: difference Fourier map
Least-squares matrix: full	Hydrogen site location: inferred from neighbouring sites
*R*[*F*^2^ > 2σ(*F*^2^)] = 0.030	H-atom parameters constrained
*wR*(*F*^2^) = 0.066	*w* = 1/[σ^2^(*F*_o_^2^) + (0.0262*P*)^2^ + 0.6663*P*] where *P* = (*F*_o_^2^ + 2*F*_c_^2^)/3
*S* = 1.05	(Δ/σ)_max_ = 0.001
5210 reflections	Δρ_max_ = 0.62 e Å^−^^3^
202 parameters	Δρ_min_ = −0.39 e Å^−^^3^
0 restraints	Extinction correction: *SHELXL*, Fc^*^=kFc[1+0.001xFc^2^λ^3^/sin(2θ)]^-1/4^
Primary atom site location: structure-invariant direct methods	Extinction coefficient: 0.0062 (3)

### Special details

**Table:**

Geometry. All e.s.d.'s (except the e.s.d. in the dihedral angle between two l.s. planes) are estimated using the full covariance matrix. The cell e.s.d.'s are taken into account individually in the estimation of e.s.d.'s in distances, angles and torsion angles; correlations between e.s.d.'s in cell parameters are only used when they are defined by crystal symmetry. An approximate (isotropic) treatment of cell e.s.d.'s is used for estimating e.s.d.'s involving l.s. planes.
Refinement. Refinement of *F*^2^ against ALL reflections. The weighted *R*-factor *wR* and goodness of fit *S* are based on *F*^2^, conventional *R*-factors *R* are based on *F*, with *F* set to zero for negative *F*^2^. The threshold expression of *F*^2^ > σ(*F*^2^) is used only for calculating *R*-factors(gt) *etc*. and is not relevant to the choice of reflections for refinement. *R*-factors based on *F*^2^ are statistically about twice as large as those based on *F*, and *R*- factors based on ALL data will be even larger.

### Fractional atomic coordinates and isotropic or equivalent isotropic displacement parameters (Å^2^)

**Table:**

	*x*	*y*	*z*	*U*_iso_*/*U*_eq_	
Ge1	0.44925 (2)	0.553177 (15)	0.551768 (15)	0.01582 (6)	
Ge2	0.35670 (2)	0.459956 (15)	0.670324 (16)	0.01586 (6)	
C1	0.5900 (3)	0.65022 (16)	0.61880 (17)	0.0266 (5)	
H1A	0.6311	0.6837	0.5705	0.040*	
H1B	0.6665	0.6172	0.6675	0.040*	
H1C	0.5430	0.6983	0.6520	0.040*	
C2	0.2832 (2)	0.62288 (17)	0.47117 (16)	0.0264 (5)	
H2A	0.3153	0.6698	0.4284	0.040*	
H2B	0.2340	0.6580	0.5140	0.040*	
H2C	0.2171	0.5757	0.4308	0.040*	
C3	0.2686 (2)	0.33907 (14)	0.60806 (14)	0.0175 (4)	
C4	0.1389 (2)	0.34120 (15)	0.53599 (16)	0.0224 (4)	
H4A	0.0886	0.4014	0.5208	0.027*	
C5	0.0824 (2)	0.25622 (17)	0.48630 (17)	0.0264 (5)	
H5A	−0.0056	0.2588	0.4372	0.032*	
C6	0.1544 (2)	0.16757 (16)	0.50815 (17)	0.0256 (5)	
H6A	0.1155	0.1097	0.4742	0.031*	
C7	0.2829 (2)	0.16369 (15)	0.57958 (16)	0.0236 (4)	
H7A	0.3320	0.1031	0.5949	0.028*	
C8	0.3399 (2)	0.24892 (15)	0.62893 (15)	0.0201 (4)	
H8A	0.4285	0.2459	0.6775	0.024*	
C9	0.5064 (2)	0.42690 (14)	0.78825 (15)	0.0170 (4)	
C10	0.4757 (2)	0.41586 (16)	0.87949 (16)	0.0227 (4)	
H10A	0.3798	0.4234	0.8844	0.027*	
C11	0.5827 (3)	0.39404 (16)	0.96343 (16)	0.0255 (5)	
H11A	0.5593	0.3861	1.0247	0.031*	
C12	0.7234 (2)	0.38385 (15)	0.95799 (16)	0.0249 (5)	
H12A	0.7968	0.3701	1.0155	0.030*	
C13	0.7560 (2)	0.39384 (16)	0.86836 (16)	0.0244 (5)	
H13A	0.8521	0.3859	0.8641	0.029*	
C14	0.6489 (2)	0.41554 (15)	0.78413 (16)	0.0204 (4)	
H14A	0.6730	0.4227	0.7230	0.024*	
C15	0.2120 (2)	0.53782 (15)	0.71306 (15)	0.0182 (4)	
C16	0.2365 (2)	0.63689 (15)	0.73511 (16)	0.0222 (4)	
H16A	0.3224	0.6664	0.7276	0.027*	
C17	0.1380 (2)	0.69347 (16)	0.76790 (16)	0.0245 (5)	
H17A	0.1577	0.7606	0.7835	0.029*	
C18	0.0110 (2)	0.65232 (17)	0.77794 (16)	0.0259 (5)	
H18A	−0.0574	0.6911	0.7992	0.031*	
C19	−0.0148 (3)	0.55397 (17)	0.7565 (2)	0.0317 (5)	
H19A	−0.1011	0.5250	0.7639	0.038*	
C20	0.0841 (2)	0.49707 (16)	0.72433 (18)	0.0266 (5)	
H20A	0.0645	0.4297	0.7098	0.032*	

### Atomic displacement parameters (Å^2^)

**Table:**

	*U*^11^	*U*^22^	*U*^33^	*U*^12^	*U*^13^	*U*^23^
Ge1	0.01786 (11)	0.01507 (10)	0.01456 (11)	0.00087 (7)	0.00413 (8)	0.00019 (8)
Ge2	0.01524 (11)	0.01695 (11)	0.01510 (11)	−0.00037 (8)	0.00329 (8)	0.00050 (8)
C1	0.0321 (13)	0.0246 (11)	0.0233 (11)	−0.0078 (9)	0.0076 (9)	−0.0030 (9)
C2	0.0299 (12)	0.0282 (11)	0.0200 (11)	0.0107 (9)	0.0043 (9)	0.0031 (9)
C3	0.0166 (10)	0.0214 (10)	0.0148 (9)	−0.0012 (7)	0.0046 (8)	0.0004 (7)
C4	0.0214 (11)	0.0225 (10)	0.0217 (11)	0.0004 (8)	0.0022 (8)	0.0021 (8)
C5	0.0223 (11)	0.0319 (12)	0.0225 (11)	−0.0049 (9)	0.0011 (9)	−0.0019 (9)
C6	0.0258 (12)	0.0261 (11)	0.0259 (12)	−0.0071 (9)	0.0084 (9)	−0.0065 (9)
C7	0.0257 (11)	0.0202 (10)	0.0261 (12)	0.0005 (8)	0.0086 (9)	0.0013 (8)
C8	0.0188 (10)	0.0232 (10)	0.0182 (10)	−0.0003 (8)	0.0045 (8)	0.0028 (8)
C9	0.0187 (10)	0.0144 (9)	0.0169 (10)	−0.0015 (7)	0.0027 (8)	0.0004 (7)
C10	0.0221 (11)	0.0240 (10)	0.0226 (11)	−0.0007 (8)	0.0068 (9)	0.0019 (8)
C11	0.0320 (12)	0.0276 (11)	0.0166 (10)	0.0007 (9)	0.0056 (9)	0.0048 (8)
C12	0.0293 (12)	0.0216 (10)	0.0198 (11)	0.0020 (8)	−0.0011 (9)	0.0023 (8)
C13	0.0209 (11)	0.0254 (11)	0.0258 (12)	0.0037 (8)	0.0035 (9)	0.0027 (9)
C14	0.0203 (10)	0.0236 (10)	0.0179 (10)	0.0026 (8)	0.0060 (8)	0.0011 (8)
C15	0.0172 (9)	0.0225 (10)	0.0146 (9)	0.0008 (8)	0.0031 (7)	0.0026 (8)
C16	0.0193 (10)	0.0243 (10)	0.0227 (11)	−0.0040 (8)	0.0051 (8)	−0.0038 (8)
C17	0.0266 (12)	0.0243 (10)	0.0219 (11)	0.0004 (9)	0.0048 (9)	−0.0047 (8)
C18	0.0255 (12)	0.0315 (12)	0.0225 (11)	0.0071 (9)	0.0096 (9)	0.0010 (9)
C19	0.0214 (11)	0.0352 (13)	0.0427 (15)	−0.0027 (9)	0.0162 (10)	0.0027 (11)
C20	0.0243 (12)	0.0235 (11)	0.0345 (13)	−0.0027 (9)	0.0117 (10)	0.0006 (9)

### Geometric parameters (Å, º)

**Table:**

Ge1—C2	1.959 (2)	C8—H8A	0.9500
Ge1—C1	1.959 (2)	C9—C10	1.394 (3)
Ge1—Ge1^i^	2.4276 (4)	C9—C14	1.398 (3)
Ge1—Ge2	2.4361 (3)	C10—C11	1.390 (3)
Ge2—C9	1.957 (2)	C10—H10A	0.9500
Ge2—C3	1.957 (2)	C11—C12	1.384 (3)
Ge2—C15	1.963 (2)	C11—H11A	0.9500
C1—H1A	0.9800	C12—C13	1.379 (3)
C1—H1B	0.9800	C12—H12A	0.9500
C1—H1C	0.9800	C13—C14	1.393 (3)
C2—H2A	0.9800	C13—H13A	0.9500
C2—H2B	0.9800	C14—H14A	0.9500
C2—H2C	0.9800	C15—C16	1.393 (3)
C3—C4	1.398 (3)	C15—C20	1.397 (3)
C3—C8	1.403 (3)	C16—C17	1.390 (3)
C4—C5	1.391 (3)	C16—H16A	0.9500
C4—H4A	0.9500	C17—C18	1.386 (3)
C5—C6	1.389 (3)	C17—H17A	0.9500
C5—H5A	0.9500	C18—C19	1.383 (3)
C6—C7	1.386 (3)	C18—H18A	0.9500
C6—H6A	0.9500	C19—C20	1.391 (3)
C7—C8	1.394 (3)	C19—H19A	0.9500
C7—H7A	0.9500	C20—H20A	0.9500
			
C2—Ge1—C1	108.47 (10)	C7—C8—C3	121.03 (19)
C2—Ge1—Ge1^i^	109.73 (7)	C7—C8—H8A	119.5
C1—Ge1—Ge1^i^	110.91 (7)	C3—C8—H8A	119.5
C2—Ge1—Ge2	105.19 (7)	C10—C9—C14	117.63 (19)
C1—Ge1—Ge2	110.63 (7)	C10—C9—Ge2	121.46 (16)
Ge1^i^—Ge1—Ge2	111.715 (14)	C14—C9—Ge2	120.90 (15)
C9—Ge2—C3	109.21 (8)	C11—C10—C9	121.3 (2)
C9—Ge2—C15	107.16 (9)	C11—C10—H10A	119.3
C3—Ge2—C15	109.31 (8)	C9—C10—H10A	119.3
C9—Ge2—Ge1	112.36 (6)	C12—C11—C10	120.2 (2)
C3—Ge2—Ge1	109.04 (6)	C12—C11—H11A	119.9
C15—Ge2—Ge1	109.71 (6)	C10—C11—H11A	119.9
Ge1—C1—H1A	109.5	C13—C12—C11	119.5 (2)
Ge1—C1—H1B	109.5	C13—C12—H12A	120.3
H1A—C1—H1B	109.5	C11—C12—H12A	120.3
Ge1—C1—H1C	109.5	C12—C13—C14	120.4 (2)
H1A—C1—H1C	109.5	C12—C13—H13A	119.8
H1B—C1—H1C	109.5	C14—C13—H13A	119.8
Ge1—C2—H2A	109.5	C13—C14—C9	121.0 (2)
Ge1—C2—H2B	109.5	C13—C14—H14A	119.5
H2A—C2—H2B	109.5	C9—C14—H14A	119.5
Ge1—C2—H2C	109.5	C16—C15—C20	117.75 (19)
H2A—C2—H2C	109.5	C16—C15—Ge2	119.92 (15)
H2B—C2—H2C	109.5	C20—C15—Ge2	122.31 (16)
C4—C3—C8	118.14 (19)	C17—C16—C15	121.4 (2)
C4—C3—Ge2	120.99 (15)	C17—C16—H16A	119.3
C8—C3—Ge2	120.67 (15)	C15—C16—H16A	119.3
C5—C4—C3	120.8 (2)	C18—C17—C16	120.3 (2)
C5—C4—H4A	119.6	C18—C17—H17A	119.9
C3—C4—H4A	119.6	C16—C17—H17A	119.9
C6—C5—C4	120.2 (2)	C19—C18—C17	119.1 (2)
C6—C5—H5A	119.9	C19—C18—H18A	120.5
C4—C5—H5A	119.9	C17—C18—H18A	120.5
C7—C6—C5	119.9 (2)	C18—C19—C20	120.7 (2)
C7—C6—H6A	120.0	C18—C19—H19A	119.6
C5—C6—H6A	120.0	C20—C19—H19A	119.6
C6—C7—C8	119.9 (2)	C19—C20—C15	120.8 (2)
C6—C7—H7A	120.1	C19—C20—H20A	119.6
C8—C7—H7A	120.1	C15—C20—H20A	119.6

Symmetry code: (i) −*x*+1, −*y*+1, −*z*+1.
